# Modulation of surface physics and chemistry in triboelectric energy harvesting technologies

**DOI:** 10.1080/14686996.2019.1631716

**Published:** 2019-06-17

**Authors:** Bo-Yeon Lee, Dong Hyun Kim, Jiseul Park, Kwi-Il Park, Keon Jae Lee, Chang Kyu Jeong

**Affiliations:** a Department of Materials Science and Engineering, Korea Advanced Institute of Science and Technology (KAIST), Daejeon, Republic of Korea; b Department of Nature-Inspired Nano-convergence System, Korea Institute of Machinery and Materials (KIMM), Daejeon, Republic of Korea; c Division of Advanced Materials Engineering, Chonbuk National University, Jeonju, Republic of Korea; d School of Materials Science and Engineering, Kyungpook National University, Daegu, Republic of Korea; e Hydrogen and Fuel Cell Research Center, Chonbuk National University, Jeonju, Republic of Korea

**Keywords:** Energy harvesting, triboelectric, tribo-electrification, surface engineering, nanogenerator, 50 Energy materials, 202 Dielectrics / Piezoelectrics / Insulators, 206 Energy conversion / transport / storage / recovery, 212 Surface and interfaces

## Abstract

Mechanical energy harvesting technology converting mechanical energy wasted in our surroundings to electrical energy has been regarded as one of the critical technologies for self-powered sensor network and Internet of Things (IoT). Although triboelectric energy harvesters based on contact electrification have attracted considerable attention due to their various advantages compared to other technologies, a further improvement of the output performance is still required for practical applications in next-generation IoT devices. In recent years, numerous studies have been carried out to enhance the output power of triboelectric energy harvesters. The previous research approaches for enhancing the triboelectric charges can be classified into three categories: i) materials type, ii) device structure, and iii) surface modification. In this review article, we focus on various mechanisms and methods through the surface modification beyond the limitations of structural parameters and materials, such as surficial texturing/patterning, functionalization, dielectric engineering, surface charge doping and 2D material processing. This perspective study is a cornerstone for establishing next-generation energy applications consisting of triboelectric energy harvesters from portable devices to power industries.

## Graphical abstract

**Figure:**
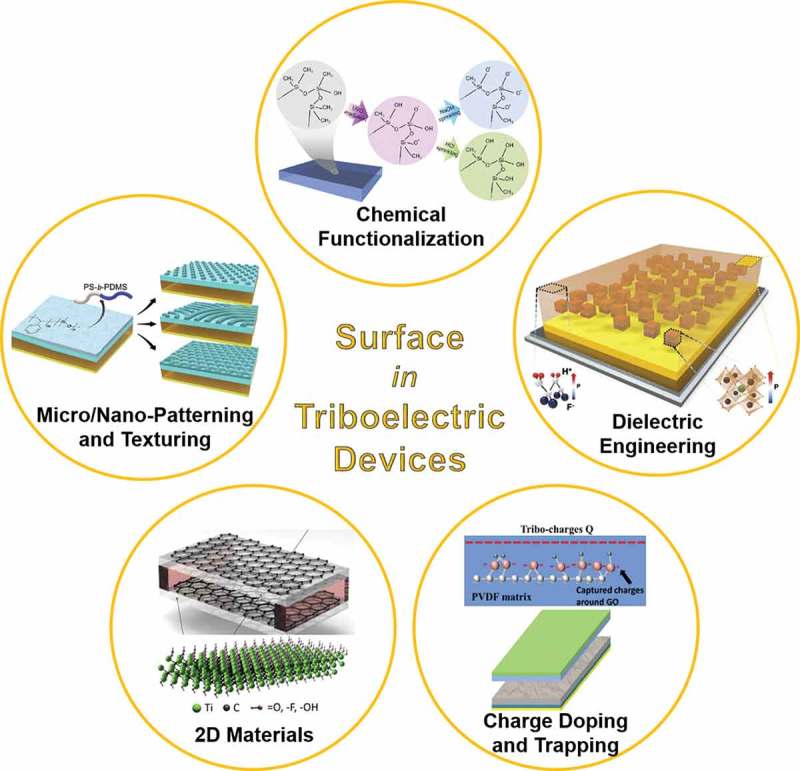

## 1. Introduction

With the advent of the Fourth Industrial Revolution and the Internet of Things (IoTs), the demand of diverse sensors that can interactively communicate with users has increased dramatically [1–4]. Unlike typical ones, these devices should be comfortable to carry or wear or even attach to curved surfaces. However, conventional battery is not suitable for the power supply of next-generation IoT sensing devices due to its several challenges such as the large volume, the inflexibility and the periodic replacement. In this respect, an energy harvesting technology is newly emerging as one solution that can substitute or supplement the existing batteries [5–10]. Especially, a flexible type of mechanical energy harvester converting the kinetic energy into the electricity has attracted much attention because it can provide the sustainable energy in isolated, indoor environment and biomechanical conditions [11–17].

A piezoelectric energy harvester is one of the ways to harvest mechanical energy, which has been widely investigated by many researchers [18–30]. When a piezoelectric device is deformed by an external mechanical stress, a dipole moment is changed inside the piezoelectric material, and thereby an electric charge is generated [31–37]. Although inorganic ceramics such as a lead zirconate titanate (PZT) or organic polymers such as polyvinylidene fluoride (PVDF) were widely used as active materials of energy harvesters, but the brittle nature and the thick thickness or the low piezoelectric coefficient caused limitations in the various applications [38–44]. In recent years, high-performance flexible energy harvesters based on composites or inorganic thin films were successfully developed with the advanced fabrication processes, which are suitable for diverse self-powered biomonitoring and biomedical sensors [45–52]. Nevertheless, energy harvesters still have some disadvantages such as restricted performance and materials limitation to be commercialized.

To extend energy harvesting technology beyond the piezoelectric energy harvester, a new type of an energy harvesting device was suggested in 2012, called triboelectric generator (TEG) or energy harvester [15]. The basic structure of the TEG based on the coupling effect of the contact electrification and the electrostatic induction is composed of two contact (friction) films with proper electrode positioning [7,53]. Note that the triboelectric effect is a well-known phenomenon that two surfaces having different triboelectric property become electrically charged during mechanical contacts [54–57]. The surface potential difference is generated by the tribocharges resulting from the contact between the surfaces having different charge affinity, thereby inducing electron flow from one to the other electrodes throughout an external circuit to keep the electrostatic equilibrium [58–61]. Based on the working principle, the output performance of TEGs is basically determined by tribocharges [62]. Therefore, considerable efforts have been thus made to increase the amount of tribocharges on the surface. Previous research approaches can be largely divided into three categories such as material pair selection [63–67], device structural design [68–73], and surface modification [74–84]. First, from the viewpoint of material pair election, all materials have the different triboelectric polarity, which means that they have the relative tendency to accept or donate surface charges during contacts [55,66]. The amount of transferred charges becomes higher with the larger difference in the triboelectric polarity for contact surfaces, and vice versa. Accordingly, two contact materials of TEGs are generally chosen far apart each other in the triboelectric series where materials are listed in the order of relative triboelectric polarity [64,67]. Furthermore, materials not listed in the typical triboelectric series can be also evaluated and considered by utilizing surface analysis equipment [66,85,86]. Another way to improve the performance is the optimization of the device structure, such as sliding-mode, rotating-type, non-contact mode, and so forth [68–73]. The other approach is physical and chemical modifications of contact surfaces. This method is an effective way to further maximize the output performance of the optimized device with material selection and structural optimization. The physical modification refers to the increase of contact surface area through the introduction of micro-/nano-structures [66,75,77–80]. This contributes to the increase of the total transferred charges. In addition, the control of surface potential through the chemical modification can make the large difference in the triboelectric polarity between contact surfaces [80–84]. Therefore, there have been diverse researches to modulate the surface potential of contact materials.

**Figure 1.**
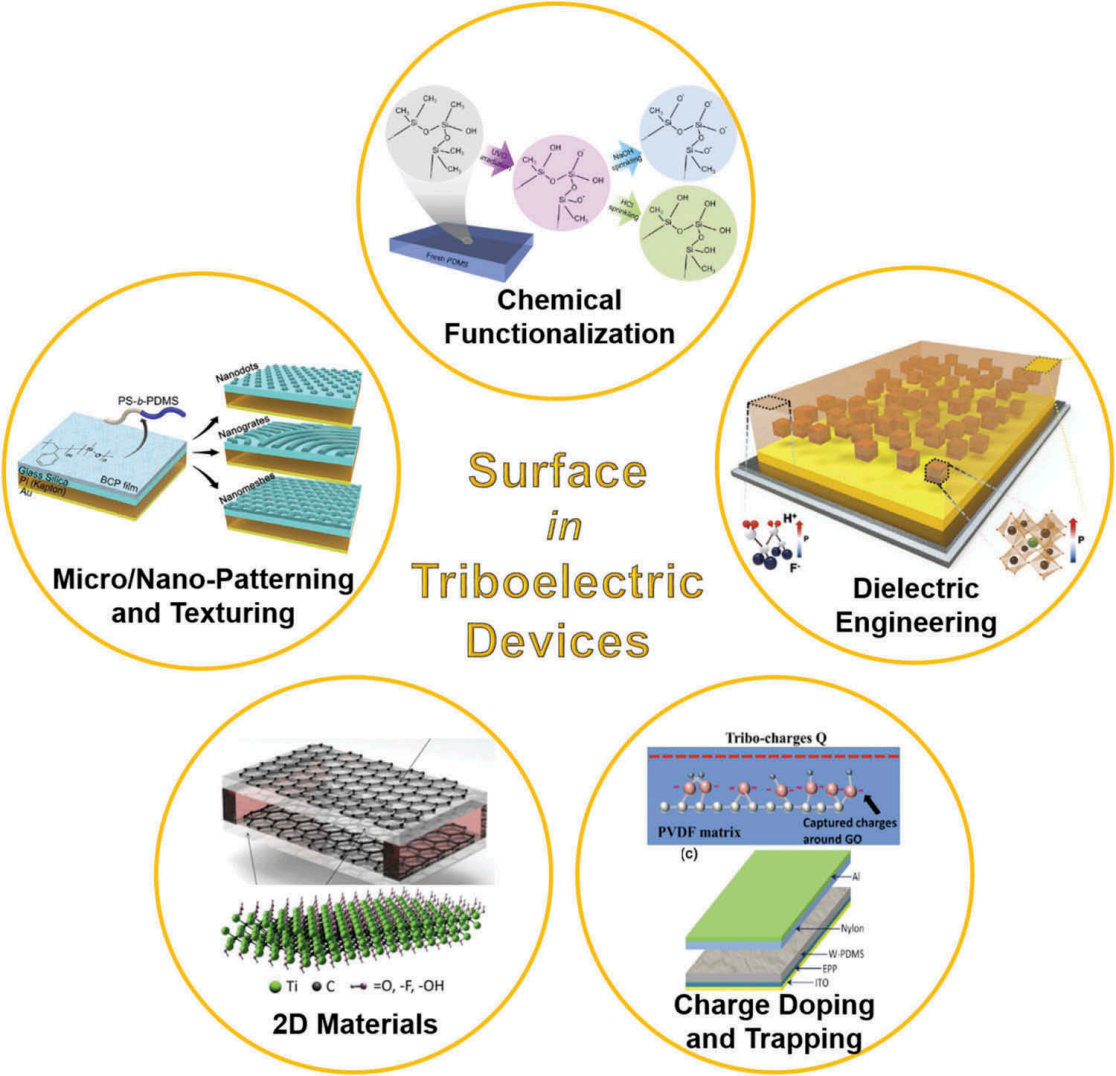
Outline of surface engineering for enhancing performance of triboelectric energy harvesting devices. Reprinted with permission from [77,100,108,110,120,123,124].

Herein, we review the important approaches and methods to modulate materials surface physically and chemically for enhancing triboelectric energy harvesting performance. The principle of triboelectric energy harvesting is first described in a representative device structure. As aforementioned, the crucial mechanism of the triboelectric energy harvesters is based on the frictional electrification by mechanical contacts or slides between two different materials, such as polymers, metals, ceramics, and even liquids [87]. To achieve record-high energy harvesting performance in the triboelectric devices, the electrified surface charges after the mechanical input should be enhanced using various physical or chemical approaches. In the early stages of triboelectric device research, the random surface morphologies were applied to the devices using intrinsic roughness or plasma etching, to name a few [3,88,89]. However, they presented serious demerits such as morphological limitation, poor controllability, unavailable materials, and so on. As shown in Figure 1, we introduce the recently reported advances in surface engineering of triboelectric energy harvesting technologies, such as surface texturing/patterning, chemical functionalization, dielectric engineering, charge doping, and two-dimensional (2D) materials processing.

## 2. Principle of triboelectric energy harvesting

**Figure 2.**
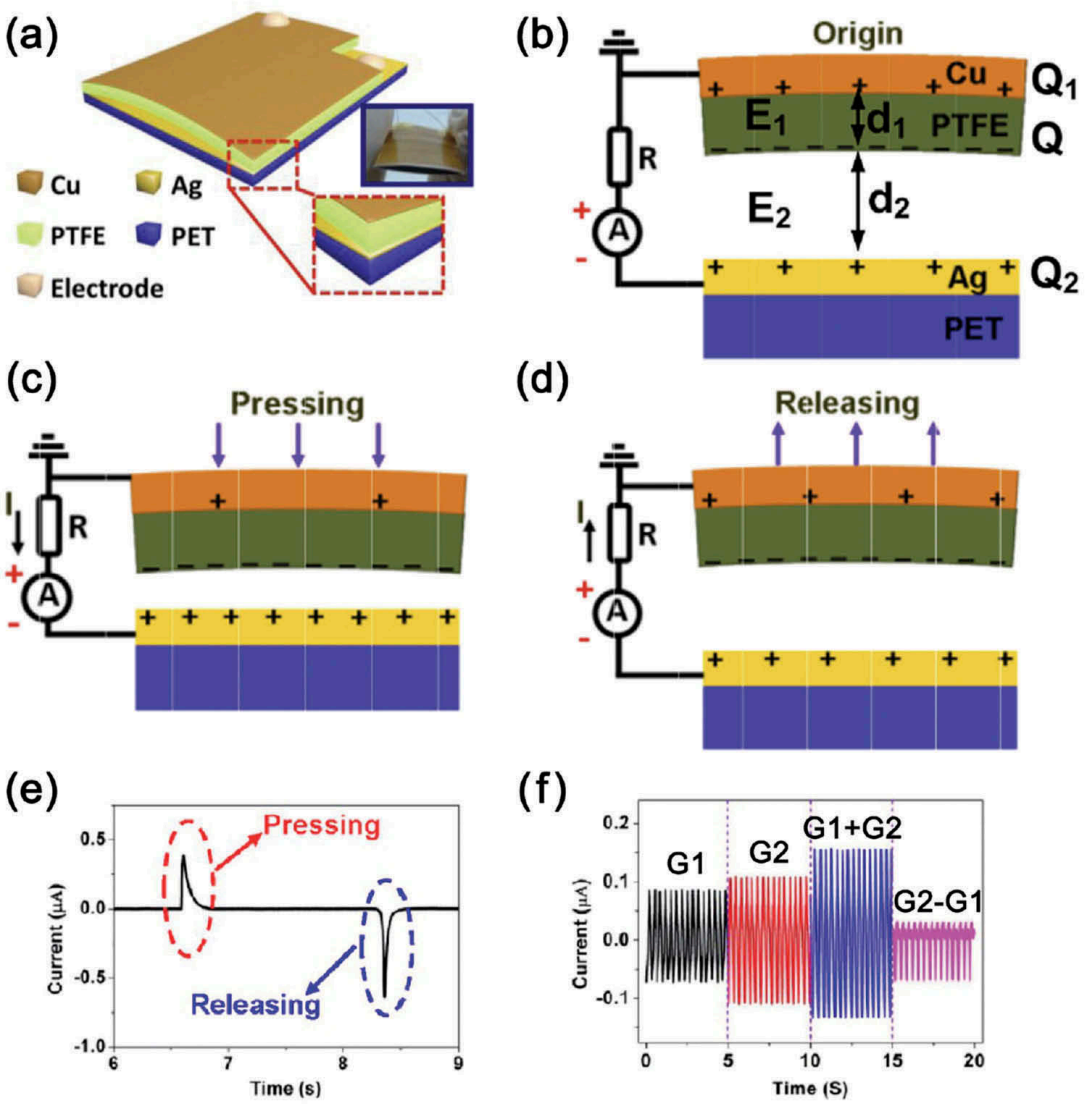
(a) Schematic illustration and photograph of a simple-structured triboelectric energy harvester of the metal-to-insulator type in contact-separation mode. Equivalent circuits of the triboelectric energy harvesting system with an external load when the device is in (b) original, (c) pressed, and (d) released states, and (e) corresponding generated current signals during the single cycle. (f) Linear superposition tests of two triboelectric generators (G1 and G2) connected each other in parallel with same and opposite polarity. Reprinted with permission from [90]. Copyright 2013 Elsevier.

Figure 2(a) shows a representative example of the triboelectric energy harvester for the metal-to-insulator in a contact-separation mode device [90]. Based on the triboelectric series, electrons (or anions) are injected from the silver (Ag) electrode to the polytetrafluoroethylene (PTFE) surface, inducing the net negative charges (*Q*) on the PTFE part. A schematic equivalent circuit of the triboelectric device with the external load (*R*) is depicted in Figure 2(b-d). Note that the device can be considered as a flat-plate capacitor with a distance-changeable air gap. The charge density of the PTFE surface is σ, that of copper (Cu) electrode is σ_1_, and that of Ag upper surface is σ_2_ (Figure 2(b)). Assuming the uniformly distributed charges and the equilibrium state, then we can derive

(1) $$\sigma_{1}=-\sigma -\sigma_{2}$$

(2) $$\sigma_{1}=-\frac{\sigma }{1+\frac{d_{1}}{d_{2}\varepsilon_{rp}}}$$

where *d*_1_ and *ε*_rp_ are the thickness and the permittivity of PTFE, respectively. We postulate that the charge *Q* is relatively stable for a long duration time. Therefore, *σ*_1_ is affected by the distance of air gap (*d*_2_). The charges between Cu and Ag electrodes should be redistributed by the change of *d*_2_, resulting in the voltage potential between the gap and the current generation through the circuit load *R*. When the triboelectric generator is pressed (Figure 2(c)), the gap distance decreases, resulting in the reduction of σ_1_ according to the Equation 2. Thus, an instantaneous positive current is generated. Once the device is mechanically released again (Figure 2(d)), the air gap reverts back to the original distance. Hence, the surface charge σ_1_ increases, corresponding to the restoration of the air gap distance *d*_2_, which produces an instantaneous negative current signal. The current signals are shown in Figure 2(e), and they can be merged when multiple devices are connected in parallel according to their polarity (Figure 2(f)). It should be mentioned that the basic working mechanisms of all triboelectric devices are similar although there are other materials types (i.e., insulator-to-insulator type) and/or diverse device structures (e.g., sliding, rotating, and single electrode modes).

## 3. Surface texturing and patterning

**Figure 3.**
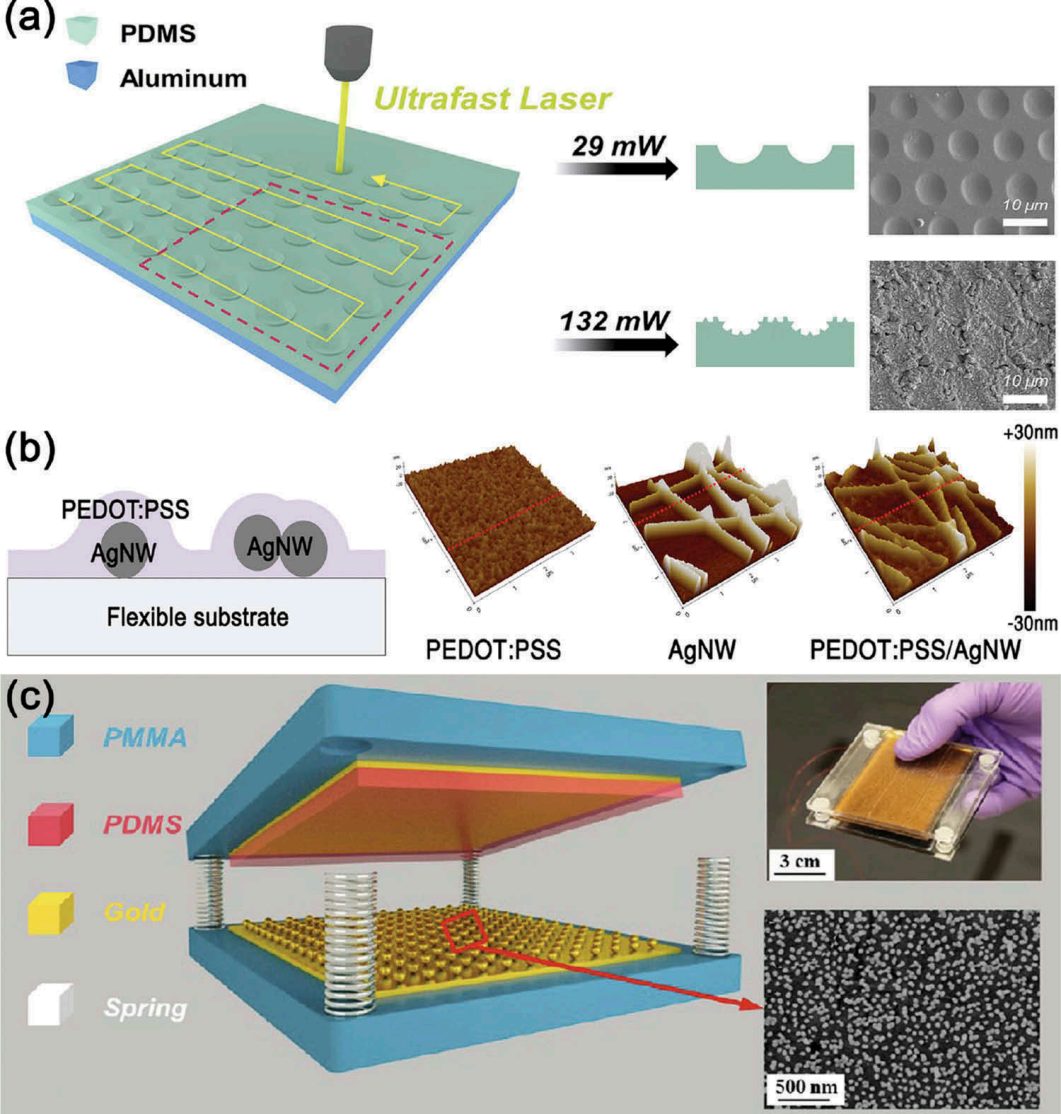
(a) Schematics of the fabrication of laser-irradiated (LI) PDMS using ultrafast laser, and corresponding scanning electron microscopy (SEM) images of the LI-PDMS at laser power of 29 mW and 132 mW. Reprinted with permission from [91]. Copyright 2017 Elsevier. (b) Schematics of the enlarged cross-sectional view of PEDOT:PSS/AgNW layer on a substrate (left). 3D topographic images of PEDOT:PSS, AgNW, and PEDOT:PSS/AgNW films (right). Reprinted with permission from [66]. Copyright 2018 Elsevier. (c) Schematic illustration, photograph, and SEM image of the Au nanoparticles-coated surface based triboelectric generator. Reprinted with permission from [75]. Copyright 2013 American Chemical Society.

Figure 3 shows the examples of methods to improve the output performance of triboelectric energy harvesters through the introduction of a micro-/nano-structures on the surface contact layers. These structures mainly contribute to enlargement of effective contact area, resulting in the enhancement of the output performance. For example, the hierarchical microstructure fabricated by ultrafast laser, as shown in Figure 3(a), resulted in the contact-area increment between friction layers, thereby increasing the output performance of the triboelectric energy harvesters [91]. Compared to the triboelectric energy harvester based on pristine polydimethylsiloxane (PDMS), the triboelectric energy harvester based on micro-structured PDMS exhibited at least two times larger output power density. Moreover, laser processing has various advantages such as direct pattering, high speed and facile adjustability [92]. Lee et al. also presented that the surface morphology can be easily modified by the presence of metallic nanowires beneath the friction layer, as shown in Figure 3(b) [66]. Consequently, the surface roughness increased more than three times, and the enlarged surface area contributed in part to the improved output power of triboelectric devices. Similarly, Figure 3(c) describes that the nanoparticle-based surface modification also plays an important role in the enhancement of the output power of triboelectric energy harvesters [75]. Physically, the bumpy surface by the synthesized and self-assembled gold (Au) nanoparticles provides a larger contact area than the flat gold thin film does.

**Figure 4.**
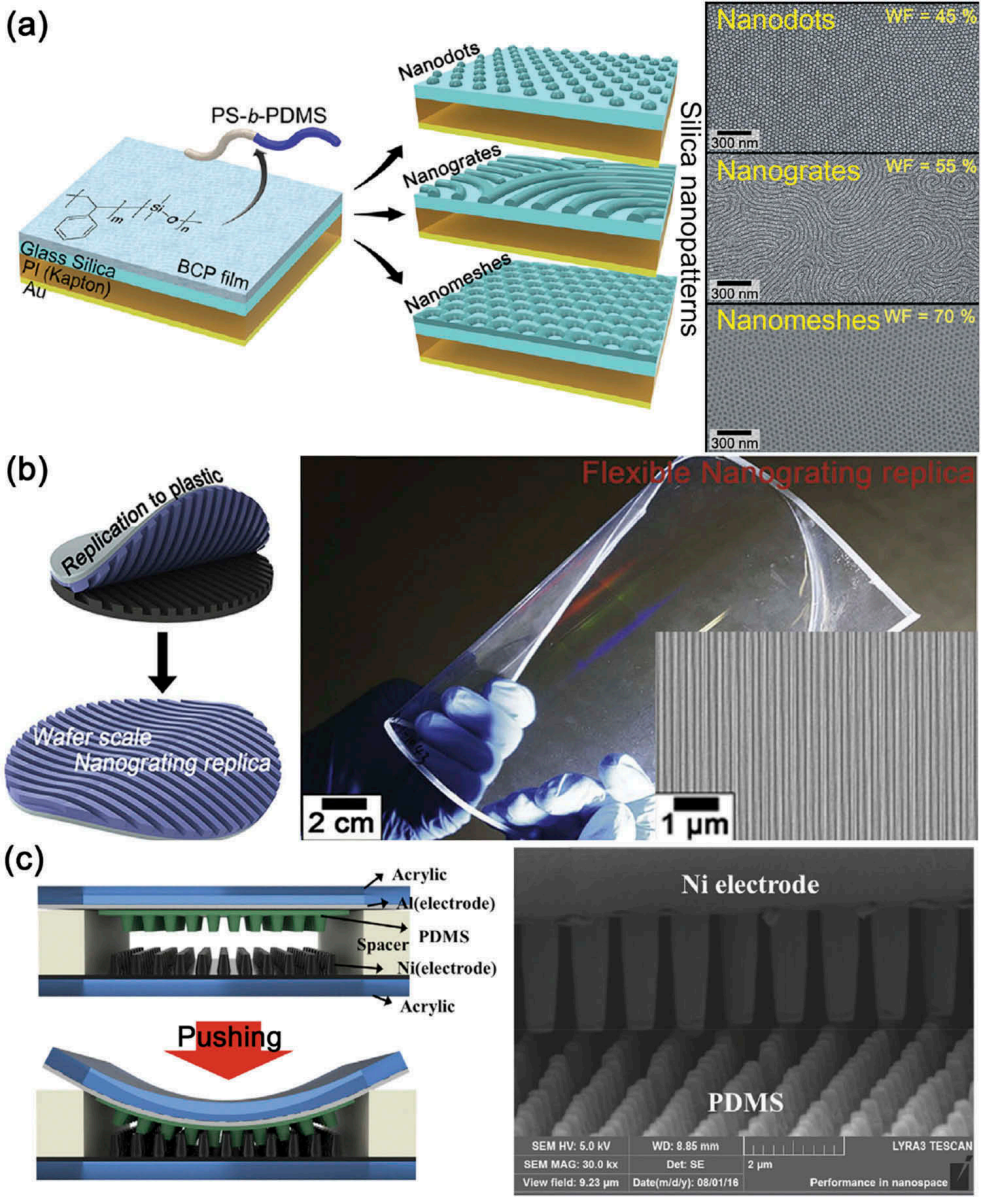
(a) Illustrated fabrication scheme of the nanopatterned surface of the block copolymer (BCP) TEG. The right panels show SEM images of BCP nanopatterned surface established by various self-assembly conditions. Reprinted with permission from [77]. Copyright 2014 American Chemical Society. (b) Schematics of nanograting replication process onto a flexible plastic substrate using ultraviolet (UV)-curable resin. The right panel is the photograph of the wafer-scale and uniform nanograting replica onto the flexible plastic substrate. The inset is the SEM image of the ultra-long and defect-free nanograting pattern of the replica on the flexible substrate. Reprinted with permission from [79]. Copyright 2017 Elsevier. (c) Schematics and SEM image of the interlocked TEG (i-TEG). Reprinted with permission from [95]. Copyright 2016 Elsevier.

Some researchers have applied highly advanced nanotechnology to the surface patterning for triboelectric energy harvesting devices. Jeong et al. demonstrated the robust and noteworthy way to triboelectric generators with nanoscale tunable surface using very controllable nanostructures via block copolymer (BCP) self-assembly processes [77]. The BCP nanopatterning and nanolithography is a very powerful approach for bottom-up nanofabrication processes to achieve various device applications [93,94]. As shown in Figure 4(a), various silica surface nanopatterns including nanodots, nanogrates, and nanomeshes on the large-area device were established according to the experimental conditions by the self-assembly of polystyrene-block-polydimethylsiloxane (PS-*b*-PDMS) BCP. In particular, they found the influence of the nanopatterning on the physical frictional events as well as contact surface area in both experimentally and theoretically. Therefore, the corresponding device performance of triboelectric energy harvesters can be also well tuned by the nanoscale surface engineering. This is the one of the representative researches for the surface nanopatterning of triboelectric devices using bottom-up nanotechnologies.

Even though bottom-up nanopatterning technologies have notable nanoscale controllability, they encumber the compatibility to the practical device fabrication because most of commercialized processes are based on top-down processes. To overcome the processing impracticality of most developments in triboelectric energy harvesting devices, the commercialized semiconducting process was adopted to achieve wafer-scale and defect-free nanoscale patterning on both rigid and flexible substrates, as presented in Figure 4(b) [79]. The polycrystalline Si nanograting patterns were fabricated by the conventional optical lithography on an 8-in Si wafer using alternative deposition of spacers and Si layers. Subsequently, the spacer sidewalls were reciprocally formed by spacer deposition and dry etching processes until the polycrystalline Si nanograting patterns were revealed. This modified spacer lithography method is called the multi-spacer pattern downscaling (MS-PaD) method. The uniform nanograting patterns can become much narrower by consuming underneath spacers and Si pattern receivers, which are the core constituents of MS-Pad method. Finally, the large-area sub-50 nm grating nanopattern was well transferred to the flexible plastic film as a replica. The scanning electron microscopy (SEM) image and the iridescent diffraction of optical photograph guarantee the uniform and well-aligned nanopatterned surface (the right panel of Figure 4(b)). The nanograting-based TEG accomplished the performance enhancement of triboelectric energy harvesting, up to 200 times higher power level, compared to the TEG of non-patterned flat surface. Moreover, they systemically demonstrated that the thickness of metal thin film on the nanopatterns can affect the triboelectric energy harvesting performance, firstly indicating the trade-off phenomena in the modulation between electrode conducting and nanopattern flattening effects. Choi et al. fabricated the nature-inspired nanopillar arrays by using the nano-imprint lithography and the electrodeposition [95]. Figure 4(c) presents the structure of nanopillar arrays-based TEG. Through the well-tailored interlocked interfaces, the contact surface area was effectively increased, resulting in the enhanced output voltage and current. Note that the fabrication of surface texturing by micro/nano- patterning and structuring have been utilized as the basis of all surface modulation approaches, which will be introduced as following sections, because the modification of surface morphology and architecture is the basic techniques in the TEGs.

## 4. Chemical functionalization and modification

**Figure 5.**
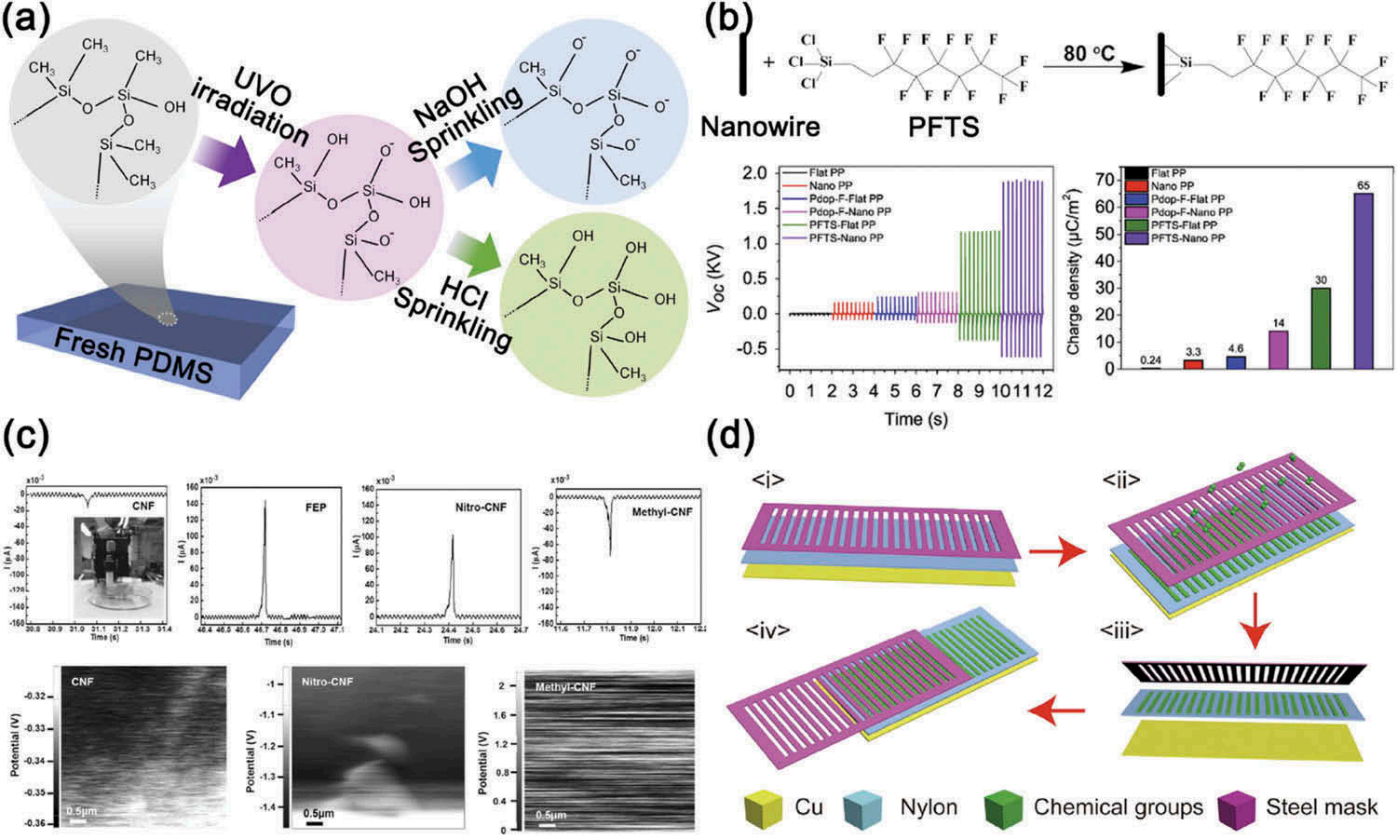
(a) The suggested mechanism of chemical elements and bonds of fresh, UV/O_3_, NaOH-, and HCl-treated PDMS surface. Reprinted with permission from [100]. Copyright 2015 Elsevier. (b) Schematics of the surface functionalization with fluorinated organic materials on the PP nanowires. Bottom panels show the voltage generation and the charge density of PP-based TEGs before and after the diverse fluorinated modifications. Reprinted with permission from [80]. Copyright 2016 Elsevier. (c) Current flow obtained from the triboelectric film of CNF, FEP, nitro-CNF, and methyl-CNF during a contact-separation cycle with Ga-In eutectic liquid (top). The scanning Kelvin probe microscopy (SKPM) surface potential mapping of pristine CNF, nitro-CNF, and methyl-CNF (bottom). Reprinted with permission from [102]. Copyright 2017 John Wiley & Sons. (d) Schematics of the reactive ion etching (RIE) and the device fabrication processes of the S-TEG-CGG. Reprinted with permission from [103]. Copyright 2017 American Chemical Society.

The surface potential of the contact materials for triboelectric energy harvesters is well-known to benefit the improvement of their output performance [96–99]. There have thus been many efforts to control surface chemical properties for the high-output power of triboelectric energy harvesters to increase the difference of triboelectric polarity between two contact surfaces. For example, polar Si-O bonds were substituted for non-polar Si-CH_3_ bonds on PDMS surface by using the ultraviolet-ozone (UVO) and the sodium hydroxide treatments, as shown in Figure 5(a). The chemically modified PDMS surface enabled the device to generate large triboelectric charges [100,101], then the modified surface-based TEG showed nearly 15-fold greater current density than the pristine TEG.

Several studies have revealed that fluorinated polymers are one of the most suitable tribo-negative contact materials for triboelectric energy harvesters due to the high electronegativity of fluorine element. Some group obtained the fluoropolymer-coated gecko feet setae-like polypropylene nanowires through a simple and modified physical vapor deposition method [80]. The surface composition with functional groups showing excellent triboelectric property improved the generation efficiency of triboelectric charges, resulting in high output performance, as shown in Figure 5(b). Figure 5(c) shows the triboelectric characteristics of nitro groups and methyl groups attached to cellulose nanofibrils (CNFs) by chemical reaction methods [102]. Cellulose shows almost relatively neutral polarity by its chemical formula, whereas the nitro group and the methyl group have an excellent electron-accepting and electron-donating properties, respectively. The TEGs composed of nitro-CNF and methyl-CNF exhibited the enhanced output power, compared with the pristine CNF-based TEGs. Furthermore, the chemical functionalization of surface contact materials can be used as a practical solution to the friction and wear due to the structural problem of sliding-mode TEGs. As shown in Figure 5(d), the positively charged nylon film partially changed to negatively charged surface through the reactive ion etching (RIE) with a metal mask [103]. The surface-modified sliding-mode TEGs exhibited high stability and strong durability owing to the chemical functionalization.

**Figure 6.**
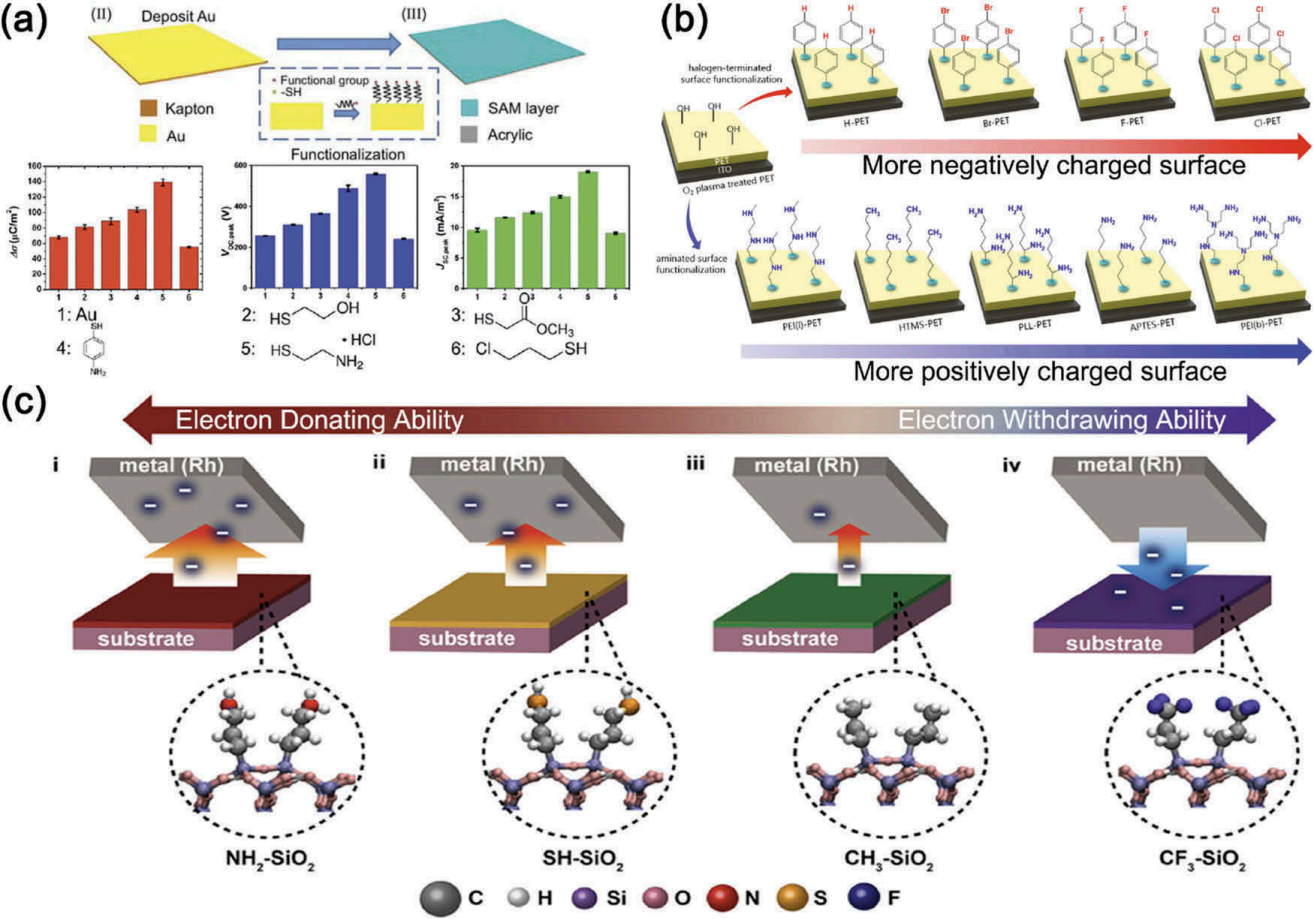
(a) A step in the fabrication process of TEG devices for the thiol-SAM modified Au films. Bottom panels present the comparisons in the transferred charge density, generated voltage, and generated current density of each SAM functionalized TEGs. Reprinted with permission from [82]. Copyright 2016 Royal Society of Chemistry. (b) Schematic illustrations of surface-functionalized polyethylene terephthalate (PET) substrates with various organic molecule head groups for negatively or positively charging. Reprinted with permission from [104]. Copyright 2017 American Chemical Society. (c) Schematic diagram showing the propensity of the triboelectrification from electron-donating to electron-withdrawing layers according to the type of surface dipoles. Reprinted with permission from [86]. Copyright 2016 American Chemical Society.

To more systematically study the effect of surface functional groups on the output performance of triboelectric energy harvesters, various attempts have been made using self-assembled monolayer (SAM) techniques. The kinds of the head group of SAMs effectively alter the surface potential of contact materials. Note that the surface potentials can be analyzed by using scanning Kelvin probe microscopy. As shown in Figure 6(a), four functional groups such as hydroxyl (-OH), ester (-COOCH_3_), amine (-NH_2_) and chloro (-Cl) were formed on the gold (Au) surface through thiol-based SAM functionalization [82]. Among them, the amine groups highly increased the surface chemical potential and thus largely promoted the charge-donating tendency. The output power of the corresponding TEG was enhanced by almost 4 times. A wide spectrum of controllable triboelectric polarity was obtained through the functionalization of surfaces with the halogen (Br, F and Cl)-containing molecules or the aminated molecules, as shown in Figure 6(b) [104]. Here, hydroxyl surface groups formed by oxygen plasma treatment played a crucial role as strong covalent bonds between the substrate and the molecules. Especially, Cl-terminated surface and branched polyethylenimine (PEI(*b*)) exhibited the most negatively and positively triboelectric properties, respectively. As a result, the Cl:PEI(*b*) contact pair-based TEG generated the large values of maximum output voltage and current density. Byun et al. even demonstrated the modified triboelectric series consisting of SAM-based contact materials. They revealed that the triboelectric property is significantly determined by surface dipoles and electronics states [86]. It was shown that the polarity and amount of triboelectric charges on the surface were well-controlled by modulating the surfaces with a wide range of electron-donating and -accepting functional groups, as shown in Figure 6(c). For instance, the positive surface dipole of CF_3_-SiO_2_ increased the surface potential, and the negative surface dipole of NH_2_, SH, and CH_3_ groups decreased the potential. In brief, these SAM-based methods for TEGs are generally simple in operation and effective for widely applicable materials.

Note that the relative polarity and strength of surface potential can be characterized by using Kelvin probe force microscope (KPFM). The measured voltage between a conductive tip of KPFM and a measured surface can reflect the surface potential of triboelectric materials. This surface potential analysis is useful mainly when the materials are not listed in the existing triboelectric series or the surface characteristics have been modified through chemical treatment [66,85,86,105].

## 5. Charge doping and trapping

**Figure 7.**
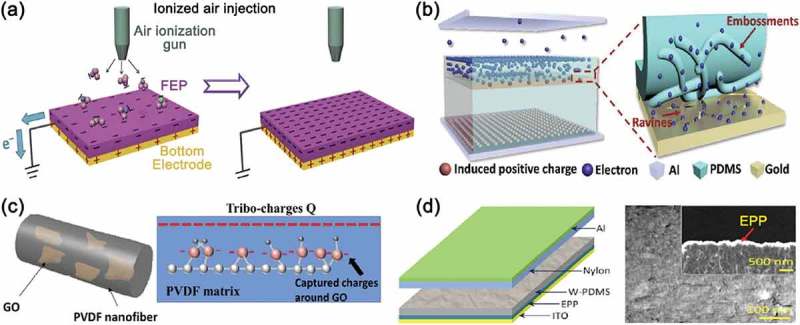
(a) Fabrication illustration of the ionic molecule-injected fluorinated ethylene propylene (FEP) film and the final charge state of the FEP film for the contact-mode TEG. Reprinted with permission from [81]. Copyright 2014 John Wiley & Sons. (b) Schemes of electron drift in the G-TEG device (left) and electron escape from PDMS to Au (right). Reprinted with permission from [107]. Copyright 2018 American Chemical Society. (c) Schemes of PVDF/GO nanofibers presenting the dispersion of GO in the nanofiber (left) and stored charges on the surface of the GO sheet (right). Reprinted with permission from [108]. Copyright 2015 Springer Nature. (d) Device design of the as-fabricated TEG improved by the hole transport layer (left) and the top-view and cross-sectional SEM images of the hole transport layer, showing ethylene glycol (EG)-PEDOT:PSS (EPP) layer coated PDMS surface (right and inset). Reprinted with permission from [110]. Copyright 2016 American Chemical Society.

In addition to the surface functionalization techniques, the method to add charges on or inside the contact materials can be a great way to enhance the output performance of triboelectric energy harvesters. The large amount of the triboelectric charges distributed on the surface of the contact materials through the charge doping or trapping can lead to a strong driving force for the high-output voltage and current. For example, the surface charge density largely increases through the direct injection of single-polarity charged particles and ions onto the contact surface, as illustrated in Figure 7(a) [81,106]. Through this simple and effective method suggested by Wang et al., a maximum power density of TEGs was improved by as much as 25 times, and the performance was maintained for several months. Furthermore, they found that the maximum achievable charge density could be more enhanced by the reduction of the thickness in the dielectric film. Trapping charges inside the triboelectric materials also causes the improvement in the output performance of triboelectric energy harvesters. This is because that the loss of triboelectric electrons can be suppressed by trapped sites. The groove structure on an Au layer fabricated by a plasma treatment acted as a trap site of triboelectric charges, as depicted in Figure 7(b) [107]. Similarly, graphene oxide (GO) sheets can be used as trap sites [108,109]. As shown in Figure 7(c), the GO, embedded in the PVDF nanofibers, played a role as charge trapping sites, which improved the output performance of the TEGs [108]. The charges trapped in the GO raised the surface potential of the PVDF nanofibers and even delayed the dissipation of the surface charges. Uddin et al. utilized a polymer mixture, poly(3,4-ethylene dioxythiophene) poly(styrene sulfonate) (PEDOT:PSS), as the charge trapping layer to increase the amount of triboelectric charges [110]. A PEDOT:PSS film, which has been widely used for a hole transport layer, was placed between a contact material and an electrode as, shown in Figure 7(d). This charge accumulator layer accelerated the flow of charges at the interface, thereby contributing to enhanced triboelectric energy harvesting.

## 6. Dielectric property engineering

Controlling the dielectric property of triboelectric materials can affect the output performance of triboelectric energy harvesters [111–116]. Triboelectric charge density on the contact materials is proportional to the maximum capacitance of the triboelectric energy harvester. It means that the charge density can be increased with the increase of the relative permittivity and the decrease of the thickness of the contact materials.

**Figure 8.**
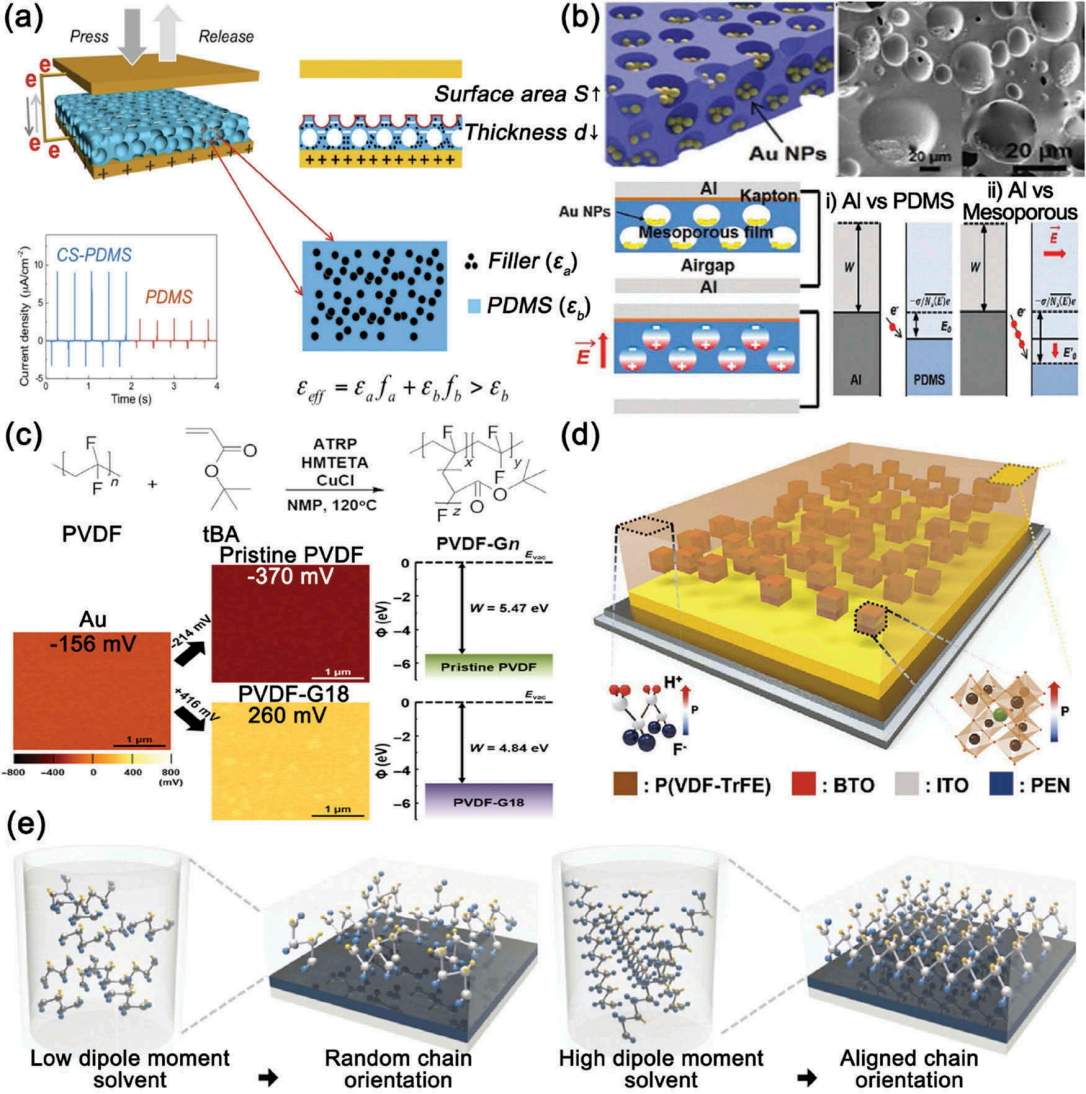
(a) Schematic illustrations of composite sponge PDMS-based TEG (CS-TEG) and its principle of performance enhancement. Reprinted with permission from [65]. Copyright 2016 American Chemical Society. (b) Schematics and SEM image of the mesoporous PDMS film filled with Au nanoparticles (top). Triboelectric charge generation mechanisms of the Au NP-embedded mesoporous triboelectric nanogenerator and schematic energy band diagram (bottom). Reprinted with permission from [118]. Copyright 2015 Royal Society of Chemistry. (c) Simple diagram of synthesis of PVDF-Gn graft copolymer for dielectric-controlled triboelectric energy harvesters (Top). The Kelvin probe force microscopy (KPFM) surface potential distribution images and work function values of pristine PVDF and PVDF-G18 films (bottom). Reprinted with permission from [119]. Copyright 2017 AAAS. (d) Schematic description of the ferroelectric composite for control dielectric properties to enhance triboelectric energy harvesting. Reprinted with permission from [120]. Copyright 2017 John Wiley & Sons. (e) Schematic illustrations of P(VDF-TrFE) solution in low dipole moment solvent and its corresponding film on a substrate (left), and P(VDF-TrFE) solution in high dipole moment solvent and its corresponding film on the substrate (right). Reprinted with permission from [121]. Copyright 2017 John Wiley & Sons.

As shown in Figure 8(a, b), some researchers put the nanoparticles with high permittivity inside the contact materials to increase the dielectric constant of materials [65,117,118]. Chen et al. used the dielectric nanoparticles such as SiO_2_, TiO_2_, BaTiO_3_, SrTiO_3_ filling into PDMS matrices to increase the permittivity of contact materials [65]. Among them, the TEGs using SrTiO_3_ produced the highest output voltage due to its highest relative permittivity of 300. Accompanying with modulating relative permittivity, they also effectively reduced the thickness of contact materials by forming pores which were fabricated by mixing and removing NaCl salt particles. The output voltage and current density were improved with increasing porosity. They showed the maximum value at the volume ratio of 15% presumably because the relative permittivity also decreases with the increase of the porosity. Chun et al. also developed the high-power TEG based on the Au nanoparticles-embedded porous film [118]. They synthesized a copolymer to increase the dielectric constant of contact materials. As shown in Figure 8(c), PVDF was successfully incorporated with poly(tert-butyl acrylate) (PtBA) through the atom-transfer radical polymerization [119]. The PtBA composed of the functional groups containing π-bonding and polar characteristics enhanced the dipole moment of contact materials, resulting in the improved performance of TEGs. Additionally, the aligned dipoles by poling the contact materials highly spurred the charge-accepting characteristics of contact materials, thereby further increasing the output power by 20 times. Seung et al. utilized both the effects of the relative permittivity and the polarization on the output power of TEGs. As highlighted in Figure 8(d), the poled ferroelectric P(VDF-TrFE) copolymer mixing with high dielectric BaTiO_3_ was used as the contact material [120]. As aforementioned, both of electrically manipulated polarization and high dielectric property could induce a very strong surface potential on contact materials. As a result, the TEGs based on this poled P(VDF-TrFE):BaTiO_3_ showed 150 times higher output power than typical PTFE-based TEGs. In addition to electrical poling method, applying a high dipole moment solvent can be another way to align dipoles in contact materials. The end-to-end chain length and the dipole alignment were enhanced by a high dipole moment solvent such as dimethyl sulfoxide (DMSO), as indicated in Figure 8(e) [121]. Hence, the P(VDF-TrFE) polymer dissolved in the DMSO had a relatively high charge-accepting ability, leading to the output enhancement of triboelectric energy harvesters.

More notably, the shift of effective work function and the corresponding electron transport which had been insisted in the previous reports about ferroelectric (highly dielectric)-induced triboelectric enhancement [111,118] has been denied in reality [57,122]. According to the very recent study, the performance enhancement is almost due to the induction driven by piezoelectric charges [122]. In fact, the correlation between dielectric properties and triboelectric energy harvesting signals is not clearly unveiled, yet. It should be more investigated.

## 7. Surface with two-dimensional (2D) materials

2D materials are ultrathin nanomaterials which are composed of a few atomic layers, e.g., graphene. Therefore, these specially classified materials can wrap universal interfaces and surfaces with conformal coverage, which can bestow totally different physical and chemical properties upon the original material surface. In other words, adopting 2D materials would result in any strategies mentioned above.

**Figure 9.**
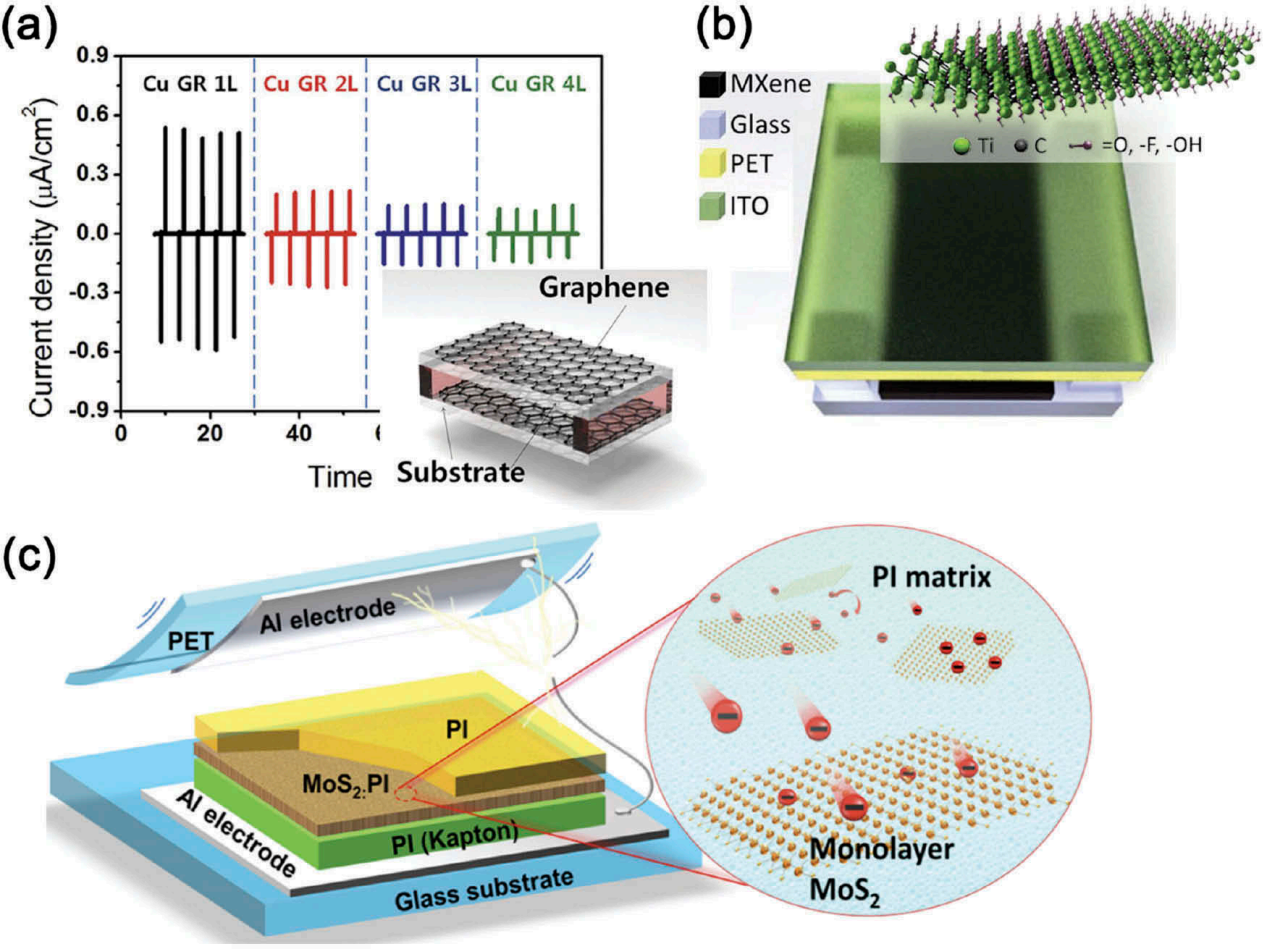
(a) Output current density generated by the Cu foil-grown 1L-, 2L-, 3L-, and 4L-stacked graphene-based TEG under same compressive force. Inset: a schematic illustration of graphene-based transparent TEG device with a spacer structure. Reprinted with permission from [123]. Copyright 2014 John Wiley & Sons. (b) Schematics of MXene TEG (MXene/glass for the bottom electrode) with an air gap between top and bottom electrodes. ITO stands for indium tin oxide and PET for polyethylene terephthalate. Inset: illustration of Ti_3_C_2_T_x_ MXene structure. Reprinted with permission from [124]. Copyright 2018 Elsevier. (c) Device structure of the TEG made by the MoS_2_ monolayer films (left). Schematics of the electron transfer from the PI matrix to the MoS_2_ monolayers (right). Reprinted with permission from [125]. Copyright 2017 American Chemical Society.

Figure 9(a) displays the energy harvesting performance generated by graphene-based TEGs [123]. Kim et al. demonstrated the flexible transparent graphene-based TEG device using monolayer (1L), bilayer (2L), trilayer (3L), and quadlayer (4L) graphene synthesized on the Cu foils [123]. They provide that the output performance of 2D material-based TEG could depend on the number of graphene layers in terms of the deviation in their work function and friction, which stems from different electronic configurations among different layer stacking features. Dong et al. synthesized an emerging family of 2D layered transition metal carbides and/or nitrides, called MXenes, and applied them to TEG devices, as displayed in Figure 9(b) [124]. MXene materials, which can be modulated by tuning the composition and the functional groups, are conducting matter and exhibit high electronegativity resulting from fluorinated groups. Wu et al. introduced 2D molybdenum disulfide (MoS_2_) monolayer sheets inside the triboelectric contact materials, as presented in Figure 9(c) [125]. Similar to the reduced GO sheet, the MoS_2_ monolayer has the electron-accepting property [109]. Furthermore, the interface trap states are distributed in the band gap because of the intrinsically large bandgap energy. Owing to the 2D MoS_2_ monolayer, the TEG exhibited 120 times larger power density compared with the pristine TEG. According to these examples, 2D materials have been regarded as new approaches to modulate universal surface for triboelectric device applications [126].

## 8. Concluding remarks and perspectives

In this review, we have taken account of the representative approaches of physical and chemical surface engineering to enhance triboelectric energy harvesting performance, such as surficial texturing/patterning, chemical functionalization, dielectric engineering, intentional charge doping and 2D material processing, after summarizing the working principle of triboelectric energy harvesting devices. Although the triboelectrification has been a very well-known and fundamental phenomenon to everyone in our life since ancient Greek era, the microscopic mechanisms of tribology in many cases are still ambiguous and dim. Nonetheless, recent developments for triboelectric energy harvesting have demonstrated tremendous advances in power performance through designing device structures and circuit engineering. Due to superficial and incompatible studies between tribology sciences and triboelectric devices, the next-generation research for triboelectric devices is now staggering under the burden of hasty commercialization. To surmount the significant restriction, we need to flash back the scientific basics of tribology and electrification on certain materials surface. Hence, reminding previously reported surface engineering approaches is considerably meaningful. In addition, it is helpful to face the mechanical problem caused by wear and abrasion without vague evasions [127]. This review has highlighted representative studies, not all researches for surface engineering of triboelectric devices. Note that some aforementioned classifications of surface engineering might overlap. Notwithstanding, our review could provide important resources to shed a light on future breakthroughs in this research field by concentrating on surface modulation because most previous review reports have focused on triboelectric device structures, performance and applications. This perspective will turn into the most sought-after outlook in the development of high-performance and practical triboelectric energy harvesting and sensor devices toward commercialization someday. Ultimately, such the development will be expected to provide sustainable power to the next-generation applications from smart IoT sensors to biomedical devices.
